# Investigation of Electrochemical, Optical and Thermal Effects during Flash Sintering of 8YSZ

**DOI:** 10.3390/ma11071214

**Published:** 2018-07-14

**Authors:** Mattia Biesuz, Lorenzo Pinter, Theo Saunders, Mike Reece, Jon Binner, Vincenzo M. Sglavo, Salvatore Grasso

**Affiliations:** 1 Department of Industrial Engineering, University of Trento, Trento 38123, Italy; lorenzo.pinter@alumni.unitn.it (L.P.); vincenzo.sglavo@unitn.it (V.M.S.); 2 School of Engineering and Material Science, Queen Mary University of London, London E1 4NS, UK; saunders.theo@gmail.com (T.S.); m.j.reece@qmul.ac.uk (M.R.); 3 School of Metallurgy and Materials, University of Birmingham, Birmingham B15 2TT, UK; J.Binner@bham.ac.uk; 4 Key Laboratory of Advanced Technologies of Materials, Ministry of Education, School of Materials Science and Engineering, Southwest Jiaotong University, Chengdu 610031, China

**Keywords:** flash sintering, electrochemistry, electrochemical blackening, yttria stabilized zirconia, electronic conductivity

## Abstract

This paper reports the electrochemical, optical and thermal effects occurring during flash sintering of 8 mol % yttria-stabilized zirconia (8YSZ). In-situ observations of polycrystalline and single crystal specimens revealed electrochemical blackening/darkening during an incubation period prior to flash sintering. The phenomenon is induced by cathodic partial reduction under DC fields. When using a low frequency AC field (0.1–10 Hz) the blackening is reversible, following the imposed polarity switching. Thermal imaging combined with sample colour changes and electrical conductivity mapping give a complete picture of the multi-physical phenomena occurring during each stage of the flash sintering event. The partial reduction at the cathode causes a modification of the electrical properties in the sample and the blackened regions, which are close to the cathode, are more conductive than the remainder of the sample. The asymmetrical nature of the electrochemical reactions follows the field polarity and causes an asymmetry in the temperature between the anode and cathode, with the positive electrode tending to overheat. It is also observed that the phenomena are influenced by the quality of the electrical contacts and by the atmosphere used.

## 1. Introduction

Flash Sintering (FS) is a field-assisted sintering technology where an electric field is directly applied to a green sample and the current that flows causes Joule heating [1,2,3,4,5,6,7,8,9,10,11,12,13,14,15,16,17]. The sample is heated in a furnace until it reaches a suitable temperature where the conductivity suddenly rises, producing rapid heating and allowing full densification in just a few seconds. This phenomenon, also called Flash Event (FE), takes place at the onset of the electric field and furnace temperature combination. The signatures of a FE are rapid densification [1,12], bright light emission [18,19], and a sudden electrical resistivity drop [1,7,10,20]. When using generators operating under current and voltage limits, the flash process can be divided into three stages: Stage I is the incubation period where the material is resistive and the power source works in voltage control; Stage II is the flash event, the system switches from voltage to current control and a peak power is achieved (during the FE the highest heating rates are achieved and can even exceed 10^4^ K min^−1^ [21]); and, finally, in Stage III current control is stabilised and the operating parameters are stationary.

Although the driver for the FE is the thermal runaway by Joule heating [22,23,24], in materials with a negative temperature coefficient for electrical resistivity, it is still matter of debate whether Joule heating alone can explain the observed photoemission, rapid sintering and electrical transport. Whilst some authors concluded that FS can be fundamentally explained by thermal effects [19,25,26] associated with the extremely high heating rates, others have suggested that it is due to the activation of “unconventional” phenomena induced by the electric field and current flow [27,28,29]. Different mechanisms have been proposed and, among them, localized particle surface/grain boundary melting [30,31], field-induced nucleation of lattice point defects (i.e., Frenkel pairs) and their consequent ionization causing electronic disorder [18,32].

Even though the FS mechanisms are still under debate, strong experimental evidence [33,34,35,36] has shown that the DC current flow and electric potential application interact with the defect chemistry in the cathodic region for ionic conductors like 8YSZ. In particular, the development of the so-called “electrochemical blackening” was observed in flash sintered yttria-stabilized zirconia (YSZ) [34]; it appears as a chromatic alteration of the ceramic, which is a fingerprint of partial electrochemical reduction [34]. This phenomenon typically develops starting from the cathode where the electrons injection from the metal electrode causes the electrochemical reduction. Interestingly, the cathodic region is also characterized by abnormal grain growth in fluorite-structured oxides like YSZ and gadolinium-doped ceria (GDC) [35,36,37], which suggests a marked interaction between this phenomenon, mass transport and diffusion kinetics. This has been attributed to a reduction of the activation energy for defect migration in the partially-reduced areas [28,35].

Although it has already been established in the literature that the partial reduction takes place at least during stage III of the flash process at the cathodic site, it is still not clear when and how it is initiated; in other words, it is not clear if it takes place before the flash transition. In addition, it is not clear if it contributes to the rapid densification of the material during FS. To address this lack of knowledge, the flash behaviour of 8YSZ in single crystals and polycrystalline ceramic was investigated. The samples were studied under the application of both AC and DC fields and in different atmospheres. In-situ observation of the specimens allowed identification of when and where the material truly entered the partial reduction regime.

## 2. Materials and Methods

Square single crystals measuring approximately 10 × 10 × 1 mm^3^, with the larger surfaces corresponding to the (100) and (111) crystallographic planes, were purchased from MTI Corporation (Richmond, VA, USA). Polycrystalline rectangular samples (60 × 8 × 1.6 mm^3^) were shaped by uniaxial pressing at 60 MPa using TOSOH-TZ-8YS powder (Tosoh, Tokyo, Japan) with 10 wt. % organic binder (Mobilcer C, Exxon Mobil, Irving, TX, USA). The green bodies were conventionally sintered close to full density at 1550 °C for 2 h using heating rate of 10 °C·min^−1^. Both polycrystalline and single crystal samples were cut into thin bars using a precision diamond saw; the final nominal dimensions of the specimens were 6.5 × 1.7 × 1.6 mm^3^ and 10 × 2 × 1 mm^3^ for polycrystalline specimens and single crystals, respectively.

The experimental arrangement was built-up using a custom-made power supply, set in current limit and able to work both in DC and in AC, with a limiting voltage of 50 V. The current and voltage limits used in the different experiments will be referred to as I_lim_ and ΔV_lim_, respectively. The AC experiments were carried out with the aim of exploring the limiting conditions for electrochemical blackening in the frequency (f) range 0.1–10 Hz. Two multimeters (AVSL-Mercury, MTTR01, Manchester, UK) were used to record current and voltage. Samples were connected to the electrical circuit by means of two different electrode/ceramic contacts: (i) two 0.3 mm thick Pt wires were simply wrapped around the specimen, and this contact mode is referred to as “bad contact”; (ii) two rectangular shaped, 0.2 mm thick Pt foils were wrapped around the specimen and connected to the ceramic surface by means of Pt paste (C60903P5 Gwent, Torfaen, UK), this contact mode is referred to as “good contact”. The region where the wires were wrapped (“bad contact”) or the Pt paste was applied (“good contact”) was 2–3 mm long on each side of the specimens.

The samples were placed into a custom-built electric furnace. A type K thermocouple was placed 2–3 mm from the sample to monitor the local temperature. The flash processes were carried out in air atmosphere at a temperature of 700 ± 10 °C. For the transparent single crystals, visible electrochemical blackening effects and the sample temperature evolution in its gauge length were recorded using a digital camera (Canon, Legria HF-R48, Tokyo, Japan) and an IR thermo-camera (FLIR Systems, 655sc, Wilsonville, OR, USA), both placed at about 30 cm from the furnace. The sample emissivity was assumed to be 1 for the thermal imaging camera. This value was chosen after an accurate calibration, which was carried out by comparing the temperatures read by the thermocouple placed by the sample and the thermo-camera. Videos recorded by the thermo-camera were analysed using Research IR software. Additionally, an optical fibre, placed about 3 mm from the sample and connected to a spectrometer (Avantes Starline, AvaSpec-2048, Apeldoorn, The Netherlands), was employed to collect in-situ photoemission spectra at the anodic, cathodic and central region of the sample. The spectra were analysed with PLASUS Specline software (Mering, Germany) [38]. The data of relative intensity vs. wavelength was carefully calibrated using an AvaLight-HAL, 10 W Tungsten Halogen Light Source (Avantes, Apeldoorn, The Netherlands) at a temperature of 2450 K. Whilst the majority of the experiments were carried out in air, for comparison some were undertaken in a semi-controlled inert atmosphere; a copper tube was directed towards the sample (at about 3 to 4 cm) with 8 L·min^−1^ of flowing Ar. The samples treated in Ar atmosphere are explicitly indicated in the figure captions.

**Figure 1:**
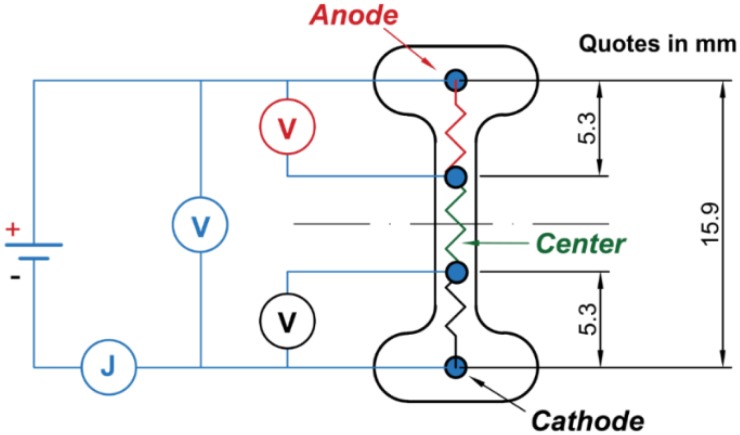
Schematic of the 4-point conductivity test on dog bone dense 8YSZ specimens.

DC-FS experiments involving 4-point conductivity measurements were carried out to measure possible variations of the potential along the gauge length. In this case, dog-bone shaped samples (which is the standard shape used in the literature for FS experiments [1,12]) were produced by uniaxial pressing the same commercial 8YSZ powder containing the same amount of organic binder as described above. After a pre-sintering treatment at 900 °C for 1 h, the samples were drilled to create four evenly spaced holes for the electric connections, Figure 1. Two Pt wires were inserted in the two cavities in the gauge length centre and covered with a paste obtained by mixing the 8YSZ powder with terpineol (Sigma Aldrich, Saint Louis, MO, USA). Subsequently, a sintering treatment at 1550 °C for 2 h was carried out to achieve fully dense samples. The specimen was connected to a DC power supply (DLM 300-2 Sorensen, San Diego, CA, USA) by two Pt wire tips, inserted in the lateral holes of the dog-bone and used as electrodes. The Pt wires were connected to two additional multimeters to record the absolute voltage between them and the electrodes, Figure 1. As indicated previously, the experiments were carried out for both “bad” and “good” contacts, the latter being achieved by spreading some Pt paste within the holes on the opposite side of the dog-bone sample, whilst the former was obtained by simply introducing a Pt wire in the hole.

Single crystals, untreated and tested entirely in a semi-controlled inert argon atmosphere (both the flash process and the cooling down to room temperature were carried out in Ar) were characterized by absorbance spectroscopy in the UV-A and visible ranges. Three spectra were collected corresponding to the electrode and the central regions. The measurements were carried out using a V-570 spectrometer (Jasco, Easton, CA, USA).

## 3. Results

The results presented in the following paragraphs mainly refer to experiments carried out on (100)-oriented single crystal specimens. Similar findings were also collected for the (111)-orientation and polycrystalline samples and they are reported in the “Supplementary Material” (Figures S1–S4).

**Figure 2:**
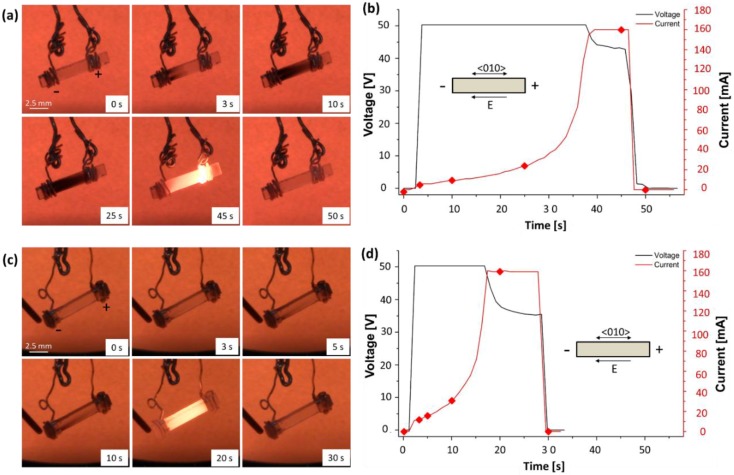
Video stills recorded using a digital camera at different intervals during a DC (ΔV_lim_ = 50 V; I_lim_ = 165 mA) FS experiment carried out with (**a**,**b**) “bad contact”; and (**c**,**d**) “good contact”. The six red dots give the electrical parameters for the corresponding video stills. The experiments were carried out in air using a (100)-orientation single crystal, with cross section of 2 mm^2^.

**Figure 3:**
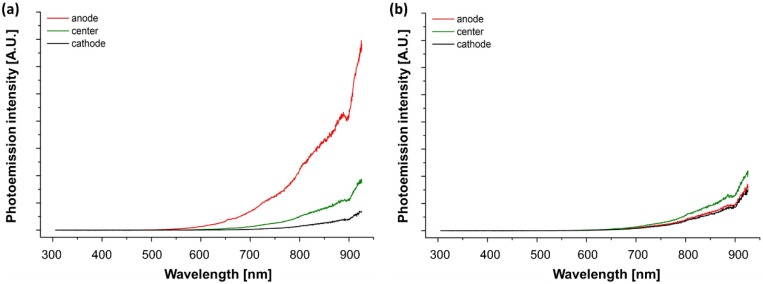
Photoemission spectra recorded in DC (ΔV_lim_ = 50 V, I_lim_ = 165 mA) FS experimental conditions when the current limit was reached. The spectra were recorded at three different points along the sample using (**a**) “bad contact” and (**b**) “good contact” connections. The experiments were carried out in an air using polycrystalline samples (cross section ≈ 2.7 mm^2^, gauge length ≈ 4 mm). The features at ≈ 655 and 890 nm correspond to instrumental artefacts.

**Figure 4:**
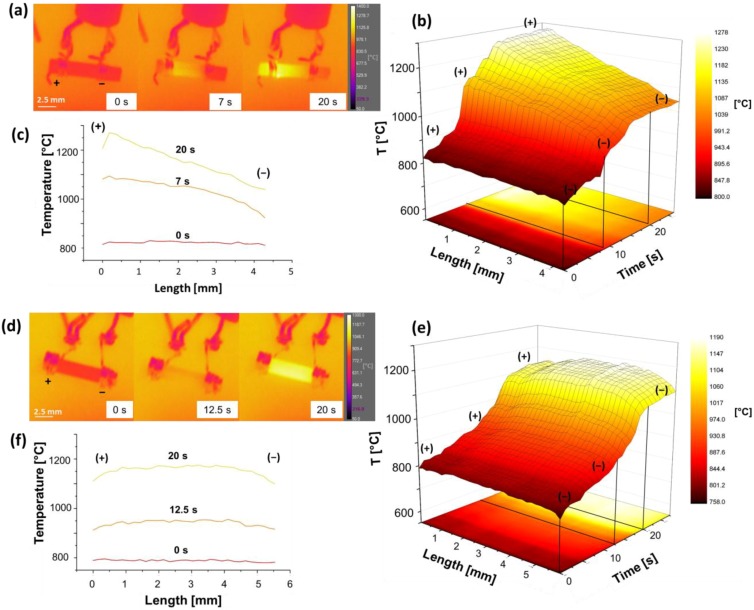
Pictures taken by the IR thermo-camera and corresponding temperature profiles at different time intervals during DC FS experiment (ΔV_lim_ = 50 V; I_lim_ = 165 mA) carried out with (**a**,**c**) “bad contact” and (**d**,**f**) “good contact”. The temperature evolution along the gauge length as a function of the treating time is also reported for (**b**) “good contact” and (**e**) “bad contact” configuration. The experiments were carried out in air using the (100) single crystal (cross section ≈ 2 mm^2^). Anode (**+**) and cathode (**−**) are indicated.

Figure 2, Figure 3 and Figure 4 refer to experiments carried out in air at T = 700 °C, ΔV_lim_ = 50 V being applied along the <010> direction and I_lim_ ranging from 160 to 170 mA. The corresponding digital camera and thermo-camera videos are reported in “Supplementary Material”, Video S1–S4. The purpose here was to exploit the transparency of the 8YSZ single crystals to observe the evolution of the electrochemical blackening during FS and how it affects the behaviour of the sample by changing the contact configuration. More specifically, Figure 2 shows the electrochemical blackening development starting from the cathodic region and the evolution of the electrical parameters upon DC-FS experiments in “bad” and “good” electrical contact. It is clearly visible how the metal-ceramic interface affected the chromatic alteration leading to the blackening of the 8YSZ single crystal, the process being visible after ≈ 3 s under “bad” contact conditions. Once the field was removed after the flash event, the sample turned back to be transparent.

A consistent photoemission difference was also observed for the two configurations once the sample reached the current limit: asymmetrical (the anode was brighter than the cathode) in the case of “bad contact” and symmetrical for “good contact” (Figure 2). Figure 3 shows the photoemission spectra recorded at three points of the sample corresponding to the anodic, cathodic and central region. The spectra confirm the observed photoemission asymmetry for the “bad contact” configuration, whilst no appreciable asymmetry appears for the “good contact”. Moreover, all spectra follow the characteristic Black Body Radiation (BBR) trend, similar to previous experiments on alumina [19] with no evidence of possible electroluminescence effects during FS as reported elsewhere [18].

Figure 4 shows the pictures taken by the thermo-camera and corresponding temperature profile along the sample during the FS experiments previously described. An evident temperature difference appears in the case of the “bad contact” configuration, the anode being ≈ 230 °C hotter than the cathode after 30 s when the current limit was about 160 mA. Conversely, no temperature asymmetry is shown for the “good contact” configuration.

**Figure 5:**
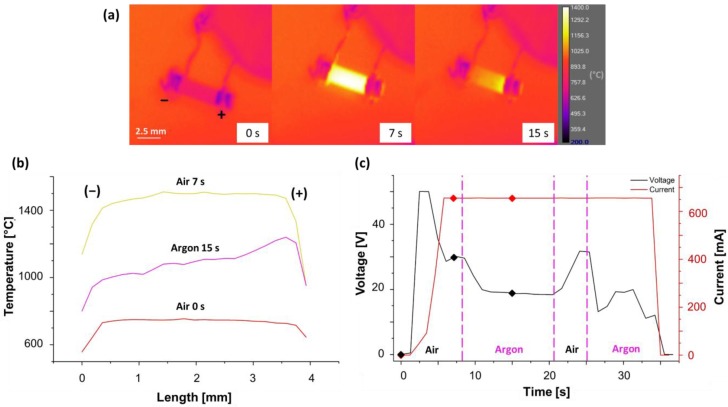
(**a**) Pictures taken with the IR thermo-camera during DC (ΔV_lim_ = 50 V; I_lim_ = 650 mA) FS experiment in “good contact” configuration and corresponding temperature profile along the samples (**b**); the evolution of voltage and current together with the different environment are shown in (**c**). The experiments were carried out with 8YSZ polycrystalline sample (cross section ≈ 2.7 mm^2^).

Figure 5 highlights the effect of the atmosphere on the flash process in the “good contact” configuration (ΔV_lim_ = 50 V, I_lim_ = 650 mA), the corresponding videos are available in “Supplementary Material”, Video S5 and S6. When Ar was blown on to the sample the electrical properties of the material changed leading to a consistent decrease in the applied voltage (Figure 5c), that is, the material became more electrically conductive despite the cooling produced by the inert gas flow. Moreover, an initial symmetrical temperature profile observed during experiments conducted in air was converted into one that was asymmetrical when performed under Ar, Figure 5b.

**Figure 6:**
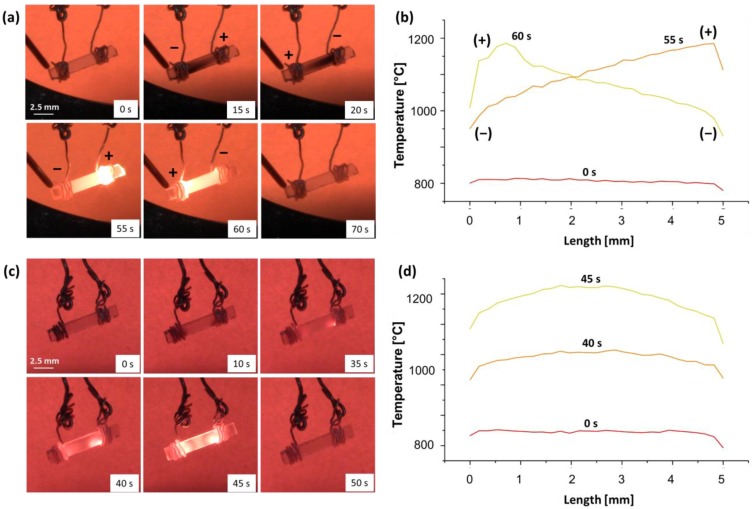
(**a**) Pictures taken by the digital camera at different times during AC (f = 0.1 Hz, ΔV_lim_ = 50 V, I_lim_ = 165 mA) FS experiment carried out with “bad contact” and (**b**) corresponding temperature profiles. (**c**) and (**d**) refer to an analogous experiment carried out using a higher AC frequency (10 Hz). The tests were carried out in air using an (100)-orientation single crystal (cross section ≈ 2 mm^2^).

The evolution of the electrochemical blackening and the temperature profiles along the sample during AC (0.1 and 10 Hz)-FS experiments are shown in Figure 6 in “bad contact” configuration (“Supplementary Material” Video S7–S11). At the lowest frequency, 0.1 Hz, the black front and the asymmetric temperature profile are periodically observed and reverse during the polarity inversion. Conversely, the application of the highest frequency resulted in limited blackening and a symmetrical temperature profile was measured.

**Figure 7:**
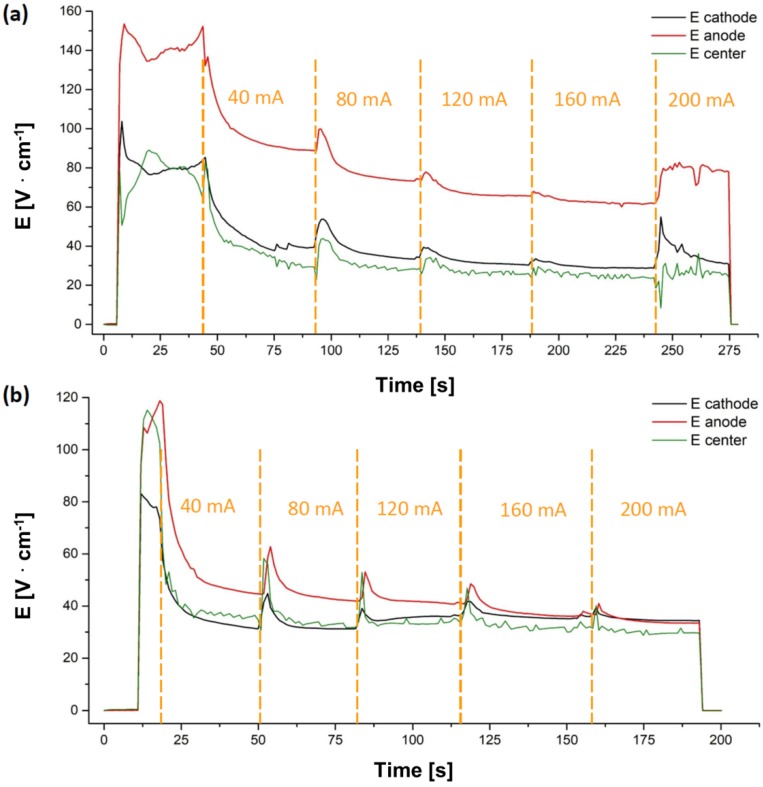
(**a**) Electrical field measured, during DC (ΔV_lim_ = 160 V) FS experiment, at the anodic, cathodic and central region along the gauge length for different current limits in “bad contact” and (**b**) “good contact” configuration. The experiments were carried out in air using polycrystalline sample (cross section = 5 mm^2^).

Figure 7 shows the results of the 4-point conductivity measurements. The experiment carried out using “bad contact” yielded a consistently different equilibrium condition for the two electrodes, the anodic region being much more resistive than the cathodic region. On the other hand, under “good contact” configuration, the electrical potentials in the different parts of the sample were quite comparable if one excludes significant electrical resistivity differences between the anodic and cathodic regions.

**Figure 8:**
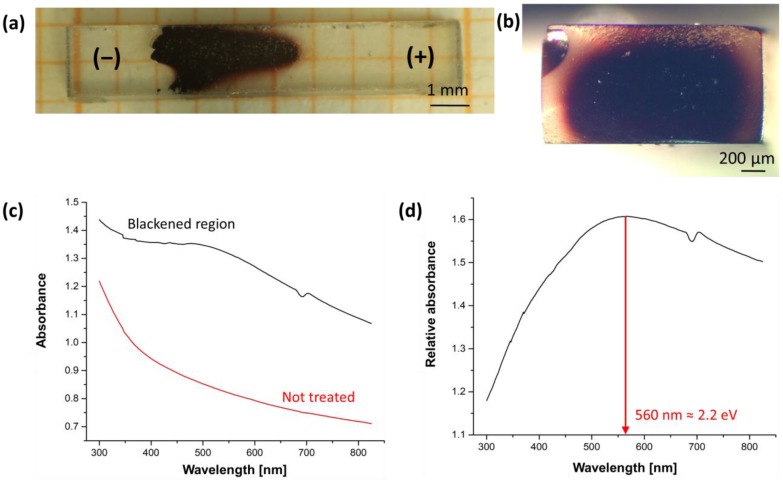
(**a**) Blackened sample (100 single crystal) after DC-FS in Ar (bad contact) along the gauge length and (**b**) in the cross section; (**c**) absorbance spectra recorded in the blackened region and in a not treated (transparent) sample; (**d**) relative absorbance in the blackened region (abs(black)/abs(not treated)). The feature at ≈ 690 nm is an instrumental artefact caused by the switching of the IR/VIS lamps.

Pictures of the specimen entirely treated in an inert Ar atmosphere (both the flash process and the cooling) are reported in Figure 8. One can observe that: (i) such treatment resulted in the electrochemical blackening remaining after cooling; and (ii) the blackening was a “bulk” phenomenon taking place mainly in centre of the sample, Figure 8b. The result of the absorbance spectroscopy characterization is presented in Figure 8c. Comparing a spectrum taken from a blackened region with one from a sample that had not been treated, it was possible to identify an absorbance band with a maximum at around 560 nm corresponding to an energy gap for an electronic transition of 2.2 eV.

Table 1 presents a summary of the main results in terms of blackening, temperature and electrical properties asymmetry achieved by changing a number of experimental parameters throughout the work.

## 4. Discussion

The blackened region generated during the incubation (Stage I, Figure 2 and Figure 6) closely resembles the so-called “electrochemical blackening” observed in doped-zirconia ceramics [33,39,40,41,42]. Such phenomenon was attributed by several authors to a partial reduction of the cations’ oxidation state [33,39,40,41,42] as a result of the application of DC electric potentials larger than 2 V [41] in reducing/inert atmospheres or when the cathode was isolated from atmospheric oxygen by sealing agents such as glasses [33].

Stoichiometric zirconium dioxide and YSZ are electronic insulators, the band gap being around 5–6 eV [43,44,45,46]. Such a band gap is much larger than the maximum energy provided by the visible electromagnetic radiation (3.1 eV). Therefore, stoichiometric crystals do not absorb visible light and are transparent (as the single crystals appear in Figure 2 and Figure 6 before the application of the electrical field). Nevertheless, once a field is applied, the material enters into an electrochemical reduction regime and new energy levels are generated within the band gap [43,44]; they are typically associated with discharge/partial discharge oxygen vacancies ($VO^{¨}+2e^{\prime }\to VO^{˙}+e^{\prime }\to VO$), also known as F and F^+^ centres [41]), which are formed during the reduction process. These defects are located about 1.5–2.3 eV below the conduction band gap [43,44]. Therefore, sub-stoichiometric zirconia absorbs visible light (1.8–3.1 eV) and loses its transparency.

The reduction mechanism is essentially based on the conversion from ionic to electronic current at the electrolyte (doped-zirconia)/metallic electrode (anode) interface. Considering the equilibrium between lattice oxygen ($O_{O}^{x}$), oxygen vacancies ($VO^{¨}$), molecular oxygen and free electrons ($e^{\prime }$):

(1) $$O_{O}^{x}↔VO^{¨}+2e^{\prime }+1/2O_{2\left(g\right)}$$

the electric current in the anodic region (+) moves the reaction towards the right, electrons being extracted from the ceramic cause the formation of $VO^{¨}$. Therefore, the anode acts as an oxygen vacancy source [33]. $VO^{¨}$ migrates towards the cathode (−) under the E-field effect where the reverse reaction takes place: molecular oxygen and vacancies are consumed by reacting with the electrons injected from the electrode and the lattice oxygen is restored:

(2) $$VO^{¨}+2e^{\prime }+1/2O_{2\left(g\right)}\to O_{O}^{x}$$

If the reaction in Equation (2) is not fast enough to sustain the ionic current flow through the electrolyte (i.e., the consumption of the oxygen vacancies by oxygen) the material enters an electrolytic reduction regime. If this is the case, according to Janek and Korte [41], a new reaction starts consuming $VO^{¨}$ and causes the formation of the blackened region starting from the cathode [33]:

(3) $$\delta VO^{¨}+ZrO_{2}+2\delta e^{\prime }\to ZrO_{2-\delta }+\delta O^{x}$$

where δ represents the amount of vacant oxygen sites in the partially reduced oxide. Such a reaction can be substantially thought as the sum of the formation of an F centre (VO)

(4) $$VO^{¨}+2e^{\prime }\to VO$$

and its incorporation in the lattice

(5) $$\delta VO+ZrO_{2}\to ZrO_{2-\delta }+\delta O^{x}$$

In fact, the discharge reaction for oxygen vacancies (Equation (4)) causes a partial reduction of the oxidation state of the cations surrounding the vacancies. It is worth noting that by combining the cathodic and anodic reactions (Equations (1) and (3)), the reduction reaction for zirconia is obtained:

(6) $$ZrO_{2}\to ZrO_{2-\delta }+1/2\delta O_{2\left(g\right)}$$

**Figure 9:**
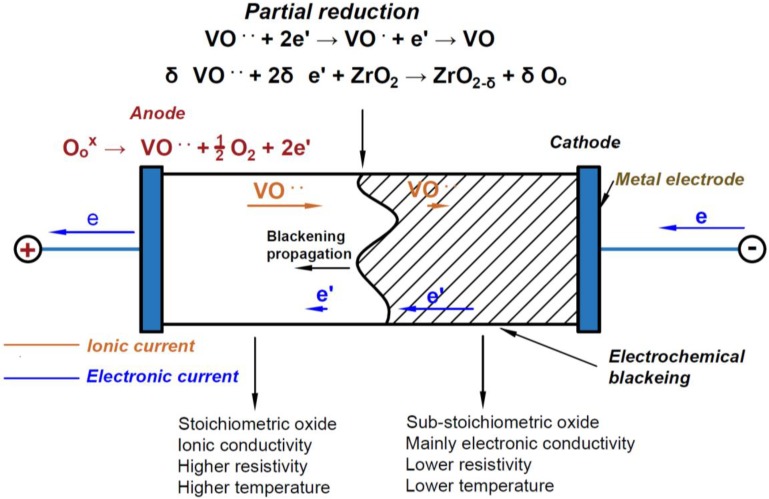
Schematic representation of the electrochemical reactions and of the blackening propagation in 8YSZ subjected to a flash sintering experiment. Drawn using [41] as reference.

A sketch summarizing the main electrochemical reaction taking place upon FS of YSZ is shown in Figure 9.

The in-situ observation of the samples during flash sintering experiments allows some important considerations:

- The observed blackening phenomenon develops from the cathode and moves to the anodic region, consistent with the well-known electrochemical blackening associated with the partial reduction of zirconia. It should also be mentioned that the more intense black colour in the centre of the specimens’ cross-section (Figure 8b) is consistent with a partial reduction: as a matter of fact, the surface was more easily re-oxidized since it was in contact with O_2_ and thus lost some of its dark colouration.
- A partial reduction (blackening) is also observed during FS experiments when they are carried out in air. Therefore, it can be observed that during the process YSZ always enters into an electrochemical reduction regime. This is a general consideration, which is true although no evident blackening is observed on the samples cooled down to room temperature: the ceramic after the flash process is quickly re-oxidised and hence loses the black colour, as observed in Figure 2.
- The blackening process does not involve only small regions close to the cathode, but extends to wider portions in the ceramic sample.
- The blackening process only takes a few seconds to develop and does not just take place during stage II or III. Therefore the material chemistry is modified by the field and current application even during the FS incubation. In other words, it is insufficient to explain the overall sample evolution upon DC-flash sintering, that is, the densification and electrical resistivity drop, without considering this phenomenon.
- The partial reduction is also evident when using AC with frequencies of 0.1 to 1 Hz, although it becomes negligible when f ≥ 10 Hz (see Figure 6 and the “Supplementary Material”).

Interestingly, the blackening/reduction development from the cathode towards the anode also appears to be associated with a thermal—electrical asymmetry during flash sintering. When the reduction (blackening) is more evident (“bad contact”) or it is promoted in an inert (Ar) atmosphere, the anodic region becomes more resistive than the cathodic, Figure 7, and, therefore, also hotter (Figure 2 and Figure 5). Anode overheating has been described before upon FS [47], but here, for the first time, it is correlated with the visible electrochemical effects. As a matter of fact, one can point out that the blackening development substantially modifies the electrical properties of the material; the cathode (where blackening starts and is more evident) being less resistive and colder than the anode. This can likely be attributed to the activation of electronic semi-conductivity in the blackened regions; when the oxide is reduced, O^2−^ ions are removed from the lattice and form O_2(g)_, thus leaving electrons in the ceramic [48]. In the case of YSZ, such electrons are mainly localized in the oxygen vacancies which are partially discharged [41]. Nevertheless, these defects are located only 1.5–2.3 eV [43,44] below the conduction band and the material behaves as a n-type semiconductor.

The atmosphere-dependent conductivity shown in Figure 5 is consistent with n-type electronic conduction in the flash regime. As a matter of fact, one can observe that the conductivity increases when the O_2_ concentration is lower, as demonstrated by the experiments involving the blowing of the Ar across the specimen, thus partially making the atmosphere less oxidising. Such behaviour is a typical fingerprint of n-type electronic conductivity [48,49]. From Equations (1) and (7),

(7) $$O_{O}^{x}+2h^{˙}\to VO^{¨}+1/2O_{2\left(g\right)}$$

where $h^{˙}$ are the electron holes, one can define the electronic defects concentration as:

(8) $$\left[e^{\prime }\right]={K_{1}}^{-1/2}{\left[VO^{¨}\right]}^{-1/2}P_{O_{2\left(g\right)}}^{-1/4}$$

(9) $$\left[h^{˙}\right]={K_{2}}^{1/2}{\left[VO^{¨}\right]}^{1/2}P_{O_{2\left(g\right)}}^{+1/4}$$

where *K*_1_ and *K*_2_ are the equilibrium constants for the reactions in Equations (1) and (7). The oxygen vacancies concentration depends on the yttrium content and it is essentially constant; for this reason ionic conductivity in YSZ does not change over a wide range of oxygen partial pressures [49]. Conversely, the free electrons concentration increases by decreasing $P_{O_{2\left(g\right)}}$ (electron holes, $h^{˙}$, show the opposite trend). Therefore, n-type conductivity is expected to increase when $P_{O_{2\left(g\right)}}$ is reduced, as observed in Figure 5. Consequently, the trigger for flash sintering under DC (or low frequency AC) field appears to have a double origin: (i) a thermal runaway of Joule heating; and (ii) the activation of electronic conduction through a partial reduction of the oxide.

Finally, it is worth discussing how the different operating conditions influence the partial reduction development, the blackened regions and the consequent electrical and temperature asymmetries. To understand the effect of the operating conditions on the reduction process one should start by analyzing the reaction in Equation (2), which represents the cathodic reaction not associated with any reduction (it does not change the oxidation state of the cations). The latter process is, conversely, represented by the reaction in Equation (3), which takes place when the rate of Equation (2) is not sufficient to sustain the current flow. The rate of Equation (2) depends on the oxygen partial pressure, O_2_ being a reagent: lower oxygen partial pressure slows down Equation (2) and promotes the reduction. This is consistent with the strong asymmetry (associated with partial reduction) observed by passing Ar onto the specimen (Figure 5). Moreover, the reaction in Equation (2) involves electrons (provided by the metal electrode), oxygen vacancies (in the ceramic) and molecular oxygen (from the atmosphere). Therefore, the reaction takes place at the interfaces between the ceramic and the electrode. In other words, the rate of reaction in Equation (2) is proportional to the interface area between Pt and YSZ. This area is very limited in the “bad contact” configuration, thus hindering the reaction in Equation (2) and promoting the reduction (Figure 2). Conversely, when a “good contact” is present, a larger metal/ceramic interface is obtained, and this accelerates the non-reducing reaction.

## 5. Conclusions

Electrochemical blackening develops during flash incubation in 8YSZ when the experiments are carried out at DC or low-frequency AC (<10 Hz). Its origin is associated with a partial reduction of the ceramic, which causes an electrical resistivity reduction. Therefore, it participates as a co-triggering mechanism to the flash transition, very likely through the activation of n-type electronic conductivity. Thus, DC or low-frequency AC field application allows switching from ionic to electronic conductivity in 8YSZ.

Moreover, the asymmetric nature of the partial reduction causes the development of significant electrical conductivity differences between the anodic and cathodic regions, the latter becoming more conductive. Such a phenomenon leads to thermal gradients along the gauge length during flash sintering, their magnitude strongly depending on the flash operative conditions, such as the treatment atmosphere and the quality of the electrical contact.

## Figures and Tables

**Table 1:** Summary of key results obtained in terms of blackening, temperature and conductivity asymmetry. The sample type is identified by a superscript: ^a^ = 100 single crystal; ^b^ = 111 single crystal; ^c^ = poly-crystal. The voltage was limited to 50 V peak value, AC had a square waveform.

Field	Contact Type	Atmosphere	Blackening	(T_anode_ − T_cathode_)_max_ (°C)	Conductivity Asymmetry
DC	BAD	AIR	YES ^a,b,c^	110 ÷ 240 ^a,b,c^	YES ^c^
		Ar	YES ^a,b,c^	NOT MEASURED	NOT MEASURED
	GOOD	AIR	POOR ^a,b,c^	≈ 0 ^a,b,c^	POOR ^c^
		Ar	YES ^c^	205 ^c^	NOT MEASURED
AC: 0.1 Hz	BAD	AIR	YES ^a,b,c^	100 ÷ 230 ^a,b,c^	NOT MEASURED
	GOOD		POOR ^a,b,c^	≈ 0 ^a,b,c^	NOT MEASURED
AC: 1 Hz	BAD	AIR	YES ^a,b,c^	NOT MEASURED	NOT MEASURED
	GOOD		POOR ^a,b,c^	NOT MEASURED	NOT MEASURED
AC: 10 Hz	BAD	AIR	POOR ^a,b,c^	≈ 0 ^a,b,c^	NOT MEASURED
	GOOD		POOR ^a,b,c^	≈ 0 ^a,b,c^	NOT MEASURED

## Supplementary Materials

The following are available online at http://www.mdpi.com/1996-1944/11/7/1214/s1, Figure S1: (a) Pictures taken by means of the digital camera at different intervals upon a DC (ΔVlim = 50 V, Ilim = 165 mA) FS experiment carried out in “bad contact” mode and (b) temperature profiles related to the same experiment. (c) Pictures taken by means of the digital camera at different intervals upon a DC (ΔVlim = 50 V, Ilim = 165 mA) FS experiment carried out in “good contact” mode and (d) temperature profiles related to the same experiment. The experiments were carried out in air using 111 orientation single crystals (cross section ≈ 2 mm^2^); Figure S2: (a) Pictures taken by means of the digital camera at different intervals upon a DC (ΔVlim = 50 V, Ilim = 165 mA) FS experiment carried out in “bad contact” mode and (b) temperature profiles related to the same experiment. (c) Pictures taken by means of the digital camera at different intervals upon a DC (ΔVlim = 50 V, Ilim = 165 mA) FS experiment carried out in “good contact” mode and (d) temperature profiles related to the same experiment. The experiments were carried out in air using polycrystalline samples (cross section ≈ 2.7 mm^2^); Figure S3: (a) Pictures taken by means of the digital camera at different intervals upon a AC (f = 0.1 Hz, ΔVlim = 50 V, Ilim = 165 mA) FS experiment carried out in “bad contact” mode and (b) temperature profiles related to the same experiment. (c) Pictures taken by means of the digital camera at different intervals upon a AC (f = 10 Hz, ΔVlim = 50 V, Ilim = 165 mA) FS experiment carried out in “bad contact” mode and (d) temperature profiles related to the same experiment. The experiments were carried out in air using 111 single crystal (cross section ≈ 2 mm^2^); Figure S4: (a) Pictures taken by means of the digital camera at different intervals upon a AC (f = 0.1 Hz, ΔVlim = 50 V, Ilim = 165 mA) FS experiment carried out in “bad contact” mode and (b) temperature profiles related to the same experiment. (c) Pictures taken by means of the digital camera at different intervals upon a AC (f = 10 Hz, ΔVlim = 50 V, Ilim = 165 mA) FS experiment carried out in “bad contact” and (d) temperature profiles related to the same experiment. The experiments were carried out in air using polycrystalline samples (cross section ≈ 2.7 mm^2^). Video S1: Digital camera video of the DC-FS (ΔVlim = 50 V, Ilim = 165 mA) on 100 single crystal in bad contact configuration; Video S2: Digital camera video of the DC-FS (ΔVlim = 50 V, Ilim = 165 mA) on 100 single crystal in good contact configuration; Video S3: Thermo-camera video of the DC-FS (ΔVlim = 50 V, Ilim = 165 mA) on 100 single crystal in bad contact configuration; Video S4: Thermo-camera video of the DC-FS (ΔVlim = 50 V, Ilim = 165 mA) on 100 single crystal in good contact configuration; Video S5: Digital camera video of the DC-FS (ΔVlim = 50 V, Ilim = 650 mA) on polycrystalline sample in good contact configuration in different atmospheres (Air and Ar); Video S6: Thermo- camera video of the DC-FS (ΔVlim = 50 V, Ilim = 650 mA) on polycrystalline sample in good contact configuration in different atmospheres (Air and Ar); Video S7: Digital camera video of the AC-FS (ΔVlim = 50 V, Ilim = 165 mA, f = 0.1 Hz ) on 100 single crystal in bad contact configuration; Video S8: Digital camera video of the AC-FS (ΔVlim = 50 V, Ilim = 165 mA, f = 1 Hz ) on 100 single crystal in bad contact configuration; Video S9: Digital camera video of the AC-FS (ΔVlim = 50 V, Ilim = 165 mA, f = 10 Hz ) on 100 single crystal in bad contact configuration; Video S10: Thermo- camera video of the AC-FS (ΔVlim = 50 V, Ilim = 165 mA, f = 0.1 Hz ) on 100 single crystal in bad contact configuration; Video S11: Thermo-camera video of the AC-FS (ΔVlim = 50 V, Ilim = 165 mA, f = 10 Hz ) on 100 single crystal in bad contact configuration.
